# Monitoring the Early Signs of Cognitive Decline in Elderly by Computer Games: An MRI Study

**DOI:** 10.1371/journal.pone.0117918

**Published:** 2015-02-23

**Authors:** Enikő Sirály, Ádám Szabó, Bernadett Szita, Vivienne Kovács, Zsuzsanna Fodor, Csilla Marosi, Pál Salacz, Zoltán Hidasi, Viktor Maros, Péter Hanák, Éva Csibri, Gábor Csukly

**Affiliations:** 1 Department of Psychiatry and Psychotherapy, Semmelweis University, Budapest, Hungary; 2 Semmelweis University, Magnetic Resonance Imaging Research Center, Budapest, Hungary; 3 Healthcare Technologies Knowledge Center, Budapest University of Technology and Economics, Budapest, Hungary; University of Glasgow, UNITED KINGDOM

## Abstract

**Background:**

It is anticipated that current and future preventive therapies will likely be more effective in the early stages of dementia, when everyday functioning is not affected. Accordingly the early identification of people at risk is particularly important. In most cases, when subjects visit an expert and are examined using neuropsychological tests, the disease has already been developed. Contrary to this cognitive games are played by healthy, well functioning elderly people, subjects who should be monitored for early signs. Further advantages of cognitive games are their accessibility and their cost-effectiveness.

**Purpose:**

The aim of the investigation was to show that computer games can help to identify those who are at risk. In order to validate games analysis was completed which measured the correlations between results of the 'Find the Pairs' memory game and the volumes of the temporal brain regions previously found to be good predictors of later cognitive decline.

**Participants and Methods:**

34 healthy elderly subjects were enrolled in the study. The volume of the cerebral structures was measured by MRI. Cortical reconstruction and volumetric segmentation were performed by Freesurfer.

**Results:**

There was a correlation between the number of attempts and the time required to complete the memory game and the volume of the entorhinal cortex, the temporal pole, and the hippocampus. There was also a correlation between the results of the Paired Associates Learning (PAL) test and the memory game.

**Conclusions:**

The results gathered support the initial hypothesis that healthy elderly subjects achieving lower scores in the memory game have increased level of atrophy in the temporal brain structures and showed a decreased performance in the PAL test. Based on these results it can be concluded that memory games may be useful in early screening for cognitive decline.

## Introduction

It is well documented that an aging society is a general tendency in Europe as well as in the United States (USA). The number of people belonging to the population aged 65 or over has tripled in the last 50 years and this tendency is expected to continue in the next 50 years [1]. Dementia is more frequent among women, some form of dementia occurs in 11% of men and 16% of women over the age of 71. Data on the prevalence of dementia are varying but studies are consistent in indicating the increasing prevalence of the disease in older age. About one third of people above 85 years of age are affected [1]. The health care of patients poses an increasingly more serious social and financial burden in an aging society. Total payments for health care, long-term care, and hospices for people with Alzheimer Dementia (AD) and other dementias in the USA are forecasted to increase from $203 billion in 2013 to $1.2 trillion in 2050 (in 2013 dollars) [1].

Since there is no effective treatment for dementia, the early detection of symptoms and the identification of methods for slowing the progression of the disease have been the main focus of medical research on the subject in recent years. The transitory condition between physiological aging and dementia known as ‘mild cognitive impairment’ (MCI) has gained a significant focus of interest. In MCI mild impairment of cognitive skills can be revealed by neuropsychological tests [2], while global cognitive functions and everyday activities are preserved. The clinical significance of the pre-disease conditions is based on the increased conversion rate of affected patients compared to the average. While dementia occurs annually in 1–4% of average elderly population, this rate is 10–15% in case of MCI [3,4]. However the process which later leads to dementia is started even before the symptoms of MCI. In view of the above it is understandable that several studies target the symptoms and differences from the average population that are closely linked to the development of dementia and can therefore be used to assist the early diagnosis.

At present cerebral imaging methods, especially MR imaging of the temporal brain regions and neuropsychological tests are considered to be the most sensitive tools for the early detection of risk [5]. In the latter case literature emphasizes the importance of the tests assessing visuospatial memory targeting the most frequent type of dementia, Alzheimer’s disease [6,7]. This is consistent with the fact that the neuropathological changes in Alzheimer’s dementia start in the entorhinal cortex and in the hippocampus years before the occurrence of clinical symptoms, and then they spread to further parts of the brain [8]. Hippocampus is the area where information on space and objects converges [9,10], therefore its functioning in visuospatial memory is crucial.

Long term follow-up studies suggest that subjects who achieved worse results in visuospatial memory tests such as the Paired Associates Learning (PAL) test compared to peer groups had a higher risk of developing dementia in later life [6]. Literature supports the importance of risk population screening, demonstrating that treatment, specifically cognitive training, started in pre-dementia stage prolongs the duration of this stage and subsequently the duration of independent living [11]. Several studies showed that cognitive training can produce moderate to large beneficial effects on memory related outcomes [12,13], enhance cognitive control [14] and reduce the risk of dementia [15] based on the brain plasticity [16,17]. However, it is important to note that screening for dementia and for MCI may have some negative effects, such as the risk of misdiagnoses due to the limited accuracy of the screening instrument, and the resulting stress caused by false positive diagnoses [17–19]. Therefore it is always important to emphasize toward participants in such programs, that the result of the screening is not equal to the clinical diagnosis, it is rather a recommendation to seek further professional help, and that it may be prudent to undergo detailed neuropsychological testing and neuroimaging.

The difficulty of screening arises from the fact that these neuropsychological tests (the base of diagnosis) were developed for clinical use. Therefore they are available only for a limited number of patients, since their application requires the active participation of an expert. Contrary to this, cognitive games provided by web pages dedicated to maintain and improve mental functions are accessible for a wider range of the population. The games on these web pages can entertain a participant, many of them have concurrently demonstrated a benefit on the development of various cognitive domains, therefore on maintaining mental wellbeing [20,21]. The widespread availability of these games and the fact that they don’t require extensive expertise can make them suitable for fulfilling the screening function [22]. Another issue with clinical neuropsychological testing is that in most cases, when subjects visit a psychologist or a psychiatrist, the symptoms of the cognitive decline are manifest and interfere with everyday functioning, i.e. the dementia has already developed. Unlike neuropsychological tests, cognitive games are played by healthy, well functioning elderly people, subjects who should be monitored for early signs. Additionally the games can be played regularly, daily or weekly, which make them a repetitive measurement, and thus ideal for screening.

In this study, the intention was to analyze the suitability of similar computer games in the detection of preclinical signs of later cognitive decline. The major cause of dementia is Alzheimer’s disease, where the visuospatial memory is the earliest function affected [6,7]. Therefore for the purpose of this study the well-known ‘Find the Pairs’ memory game (computer version) was chosen, since this game assesses this memory function. Based on the results of previous longitudinal follow-up studies it is understood that the volume of the hippocampus and the related structures, such as the volume of the temporal lobes, and the entorhinal cortex are the best predictors of cognitive decline and the later conversion to dementia [23–25]. Therefore the primary endpoint of the present investigation was to show correlation between the volumes of these Central Nervous System (CNS) structures and the results of the memory game. Since neuropsychological measures as the PAL test are also good predictors of pathological cognitive decline of elderly people, our secondary endpoint was to show correlations between this neuropsychological measure and the memory game.

## Methods

We followed the guidance of the STARDdem initiative to improve clarity when reporting our methods and results [26].

### Ethics statement

The experiments were conducted in full compliance with the Helsinki Declaration and all relevant national and international ethical guidelines. The research was approved by the National Ethics Committee, Budapest, Hungary. All procedures were carried out only after written informed consent was obtained from the participants. All potential participants who declined to participate or otherwise did not participate were not disadvantaged in any way by not participating in the study.

### Participants

Subjects were examined in the Department of Psychiatry and Psychotherapy, Semmelweis University, Budapest. Altogether 34 healthy subjects (between 50 and 80 years of age, Mean = 68, SD = 7.9, 73.5% females) were included in this study. All participants had applied to participate in a cognitive training program announced among general practitioners and in a Retirement Home (The study is registered at ClinicalTrials.gov, identifier is 'NCT02310620'). All subjects were able to lead independent lives. Basic demographic and neuropsychological data are summarized in **Table 1**.

**Table 1:** Results of the neuropsychological tests and correlations with the results of the memory game.

	MCI (n = 12)	HC (n = 34)	Healthy Subjects: Correlations with the memory game results			
	Mean (Std)	Mean (Std)	Trials needed to Complete		Time needed to Complete	
			Pearson partial correlation R [95% CI]	p value	Pearson partial correlation R [95% CI]	p value
Geriatric Depression Scale	3.2 (2.8)	4.9 (4.2)	0.15 [-0.21 0.47]	0.41	0.33 [-0.03 0.60]	0.07
Spielberger Trait-State Anxiety Inventory	30.6 (5.8)	38.6 (11.1)	0.26 [-0.10 0.55]	0.16	0.25 [-0.11 0.55]	0.17
Mini Mental State Examination	27.7 (1.3)	28.4 (1.1)	0.07 [-0.28 0.41]	0.68	-0.15 [-0.47 0.21]	0.41
Addenbrooke's Cognitive Examination (ACE)	80.0 (6.6)	91.0 (4.3)	-0.23 [-0.54 0.13]	0.20	**-0.50 [-0.72 -0.17]**	**0.004**
ACE: anterograde memory subscore	19.5 (4.3)	24.8 (2.6)	**-0.38 [-0.64 -0.04]**	**0.03**	-0.29 [-0.58 0.07]	0.11
Trail Making Test part A: Time	70.5 (28.3)	55.1 (23.2)	0.13 [-0.23 0.46]	0.48	0.30 [-0.06 0.58]	0.1
Trail Making Test part B: Time	174.2 (60.2)	122 (76.3)	0.30 [-0.06 0.59]	0.09	0.30 [-0.05 0.59]	0.09
Rey Auditory Verbal Test: Sum of Items 1–5	27.6 (5.8)	48.9 (8.0)	-0.09 [-0.42 0.27]	0.63	**-0.50 [-0.72 -0.17]**	**0.004**
Rey Auditory Verbal Test: Delayed Retrieval	2.4 (2.0)	10.3 (2.7)	-0.02 [-0.36 0.34]	0.93	-0.22 [-0.52 0.15]	0.23

Subjects with dementia were excluded from the study according to the Mini Mental State Examination (MMSE) scores standardized for age and education [27]. The exact cutoff scores for the MMSE in the different age and education groups are provided in **Table 2**.

**Table 2:** Mini Mental Examination Test (MMSE): cut-off scores for dementia.

Education Groups	Age groups	50–54	55–59	60–64	65–69	70–74	75–79	80–84	85 +
5–8 years	Cutoff score	23	23	23	23	23	21	21	17
9–12 years or high school diploma	Cutoff score	25	25	25	25	24	24	21	21
College experience or higher degree	Cutoff score	27	27	27	27	25	25	25	24

Subjects with mild cognitive impairment (n = 12, between 58 and 95 years of age, Mean = 76.1, SD = 11.4, 50% females) based on the Petersen criteria [2] were also excluded from the main correlational analyses, however they were also tested with the same neuropsychological battery (see results in Table 1), and within the framework of a pilot study their memory game results were compared with the healthy control group. The Petersen criteria include subjective memory complaint corroborated by an informant together with preserved everyday activities, a memory impairment based on a standard neuropsychological test, preserved global cognitive functions and finally the exclusion of dementia. It does not specify a neuropsychological test for the assessment of memory impairments, therefore we applied the Rey Auditory Verbal Learning Test (RAVLT), which is the most frequently used test based on the literature [5]. For the differentiation between MCI and healthy controls we applied a cutoff score of 1 SD under population mean standardized for age and gender. If a given subject scored under this cutoff value either in the total score or in the delayed recall subscore, he or she was put into the MCI group. These criteria are based on the recommendations of the National Institute on Aging—Alzheimer’s Association workgroups on diagnostic guidelines for Alzheimer's disease [12]. The exact cutoff scores for the RAVLT in the different age groups are provided in **Table 3**.

**Table 3:** Rey Auditory Verbal Learning Test (RAVLT): normative data and cut-off scores for Mild Cognitive Impairment (MCI).

Age group		50–59	60–69	70+
Total score (sum of trials 1–5)	Mean (SD)	47.6 (8.1)	43.4 (7.7)	37.1 (7.5)
	Cutoff score	39	35	29
Delayed Recall	Mean (SD)	9.9 (3.2)	8.8 (3.0)	7.0 (2.4)
	Cutoff score	6	5	4

Subjects with history of head trauma, epilepsy or stroke, or diagnosis of acute psychiatric disorder, schizophrenia or mania were excluded from the study.

### MR Image Acquisition and Processing

All patients underwent a routine brain MR examination, including high resolution anatomical images, which were used for further analysis. Image acquisitions were done at the MR Research Center, Semmelweis University, Budapest on a 3 Tesla Philips Achieva clinical MRI scanner equipped with an 8-channel SENSE head-coil. The high resolution, whole brain anatomical images were obtained using a T1 weighted 3 dimensional spoiled gradient echo (T1W 3D TFE) sequence. 180 contiguous slices were acquired from each subject with the following imaging parameters: TR = 9.7 ms; TE = 4.6 ms; flip angle = 8°; FOV of 240 mm×240 mm; voxel size of 1.0×1.0×1.0 mm.

Cortical reconstruction and volumetric segmentation were performed by Freesurfer 5.3 image analysis suite, which is documented and freely available for download online (http://surfer.nmr.mgh.harvard.edu/). The technical details of these procedures are described in prior publications, we made no changes to this pipeline. Briefly, image processing includes motion correction [28], removal of non-brain tissue using a hybrid watershed/surface deformation procedure [29], automated Talairach transformation, segmentation of the subcortical white matter and deep gray matter volumetric structures (including hippocampus, amygdala, caudate, putamen, ventricles) [30] intensity normalization, tessellation of the gray matter white matter boundary, automated topology correction, and surface deformation following intensity gradients to optimally place the gray/white and gray/cerebrospinal fluid borders at the location where the greatest shift in intensity defines the transition to the other tissue class [31,32]. Once the cortical models were completed, Freesurfer performed a number of deformable procedures for in further data processing and analysis. Steps included surface inflation [33], registration to a spherical atlas which utilized individual cortical folding patterns to match cortical geometry across subjects [34], parcellation of the cerebral cortex into units based on gyral and sulcal structure [30], and creation of a variety of surface based data including maps of curvature and sulcal depth. Segmentation and cortical models were checked and corrected manually on each subject, however correction showed no significant changes to the results.

### Procedures

The neuropsychological examinations were completed between 8 a.m. and 4 p.m. on weekdays. The examinations consisted of paper based and computerized neuropsychological tests (i.e. PAL test) and computer games. The tests took place in a separate well-lit room where only the patient and an examiner were present. Reference tests were also completed and evaluated according to the recommendations of the Neuropsychological Compendium [35], while the computerized tests and games were evaluated by the software. Instructions to the computer games were given by the examiner before playing.

Neuropsychological tests were administered by two previously trained medical students under the supervision of a psychologist and a psychiatrist. During the assessment of the tests the guidelines of the Neuropsychological Compendium [35] were followed. The paper based tests were evaluated by the same psychologist and a psychiatrist according to the compendium. The PAL test and the memory game were evaluated automatically by software. MR data were also analyzed and evaluated automatically by the Freesurfer and SAS software; therefore no subjective judgments were involved in the analysis of neuroimaging data. Since the assessment and evaluation of the memory game, the PAL test, and neuroimaging data were totally automatic, the bias from human judgments were low (limited only to the evaluation of the paper based tests).

Subjects with dementia were excluded from the study based on the Mini Mental State Examination (MMSE). The MMSE is a standard test; its effectiveness was proven by several studies, as a useful method in differentiating between subjects with dementia and healthy controls [5,36]. The majority of the previous studies used the cut off score of 26 for dementia. The subtasks of the test assess orientation, central executive function, rapid association formation, verbal identification ability and the ability to analyze and synthesize.

The Addenbrooke’s Cognitive Examination (ACE) was used to assess the global cognitive performance, including orientation, attention, memory, verbal fluency, verbal and visuospatial skills [37,38].

The Rey Auditory Verbal Learning Test (RAVLT) was used for the detailed assessment of memory functions based on Petersen criteria. Rey test evaluates verbal learning and memory [39]. A list of 15 words (list A) should be repeated by the subject immediately. This test is repeated 5 times. Then another list of 15 words (list B or interference list) is presented once that should be recalled. Then list A should be recalled without repeating, and then this task is repeated after 30 minutes.

The Trail Making test, Part A and Part B (number connection) [38,40,41] is used to evaluate selective attention, cognitive flexibility and executive functions. In Part A, randomly distributed numbers should be connected in numerical order, while in Part B randomly distributed numbers and letters are displayed. The subject is instructed to connect them in a pre-defined order. The time required to complete the test is the dependent variable. Part A of TMT measures attention and executive functions, while Part B is also affected by cognitive flexibility.

The results of the neuropsychological tests are summarized in **Table 1**.

All subjects completed a form in which they evaluated their own memory function and health condition; furthermore, they had to report on their recreational activities, computer and internet use, dietary habits, alcohol consumption and smoking. The Geriatric Depression Scale (GDS) was used to identify depressive symptoms [42]. Symptoms of anxiety was measured by the Spielberger Trait-State Anxiety Inventory (STAI) [43].

During the computerized tests and games subjects were seated comfortably at a distance of half a meter from the computer screen and following prior information they solved the tasks with the use of a mouse.

Visuospatial memory was measured by an implementation of the PAL test used in several neuropsychological test batteries [44]. In the PAL test windows open up in random positions on the screen after each other for 3 seconds with abstract shapes shown in one or more windows. Other windows remain blank depending on the difficulty level. When all squares were shown, the previously shown shapes appear in the centre of the screen and the participants have to decide in which window they saw that shape before. The test consist of five different levels in eight stages in total, the number of shapes increases from 1 to 8 on the different stages. The subjects had 10 trials to complete a given stage, otherwise the test ended. The arrangement of windows was asymmetrical in the test and it changed from stage to stage. [44]

The ‘Find the Pairs’ memory game requiring mainly visuospatial memory was selected from a set of computer games developed in the framework of the ‘M3W’ project (http://www.m3w-project.eu), dedicated to maintaining and measuring mental wellness among elderly people. In the beginning of the game, cards are laid face down. Two cards can be flipped face up in each turn by clicking on them. If the shapes (pictures) on the cards match, they disappear. The task is to clear all the cards from the table by finding the pairs. First there was a tutorial run on a table of 3x4 cards, afterwards participants had to complete a practice run on a table with 3x6 cards, and finally the measurement was done on a table of 4x6 cards. The position of the cards was the same for all participants. The time and clicks needed to complete the game was recorded and evaluated as part of the analysis.

### Statistical Analysis

Correlations between the results of the memory game and the size of the temporal structures were analyzed by General Linear Model Analysis (GLM in SAS 9.2) with age, gender and Total Intracranial Volume (TIV) as covariates, and are given in terms of partial correlation R. In the correlation analysis of the results of the memory game and the result of the neuropsychological test only age and gender were the covariates. Correlations with the number of stages completed in the PAL test were analyzed by Spearman Correlation, since the distribution of this variable deviated largely from the normal distribution. In order to quantify uncertainty for the correlational analyses we calculated the 95% confidence intervals.

In case of the PAL test we used the adjusted number of total trials, instead of the raw total trials since this measure takes into account that several subjects failed to complete all the stages of the test and thus have fewer opportunity to make errors. In order to calculate the adjusted measure we added the maximum score of 10 trials for each stage not attempted due to an earlier failure to the raw total trials.

This was a prospective study, since data collection was planned before the tests were performed, and the correlational analyses were planned before starting the study.

## Results

### Correlations between Temporal Structures and the Memory Game

**Fig 1:**
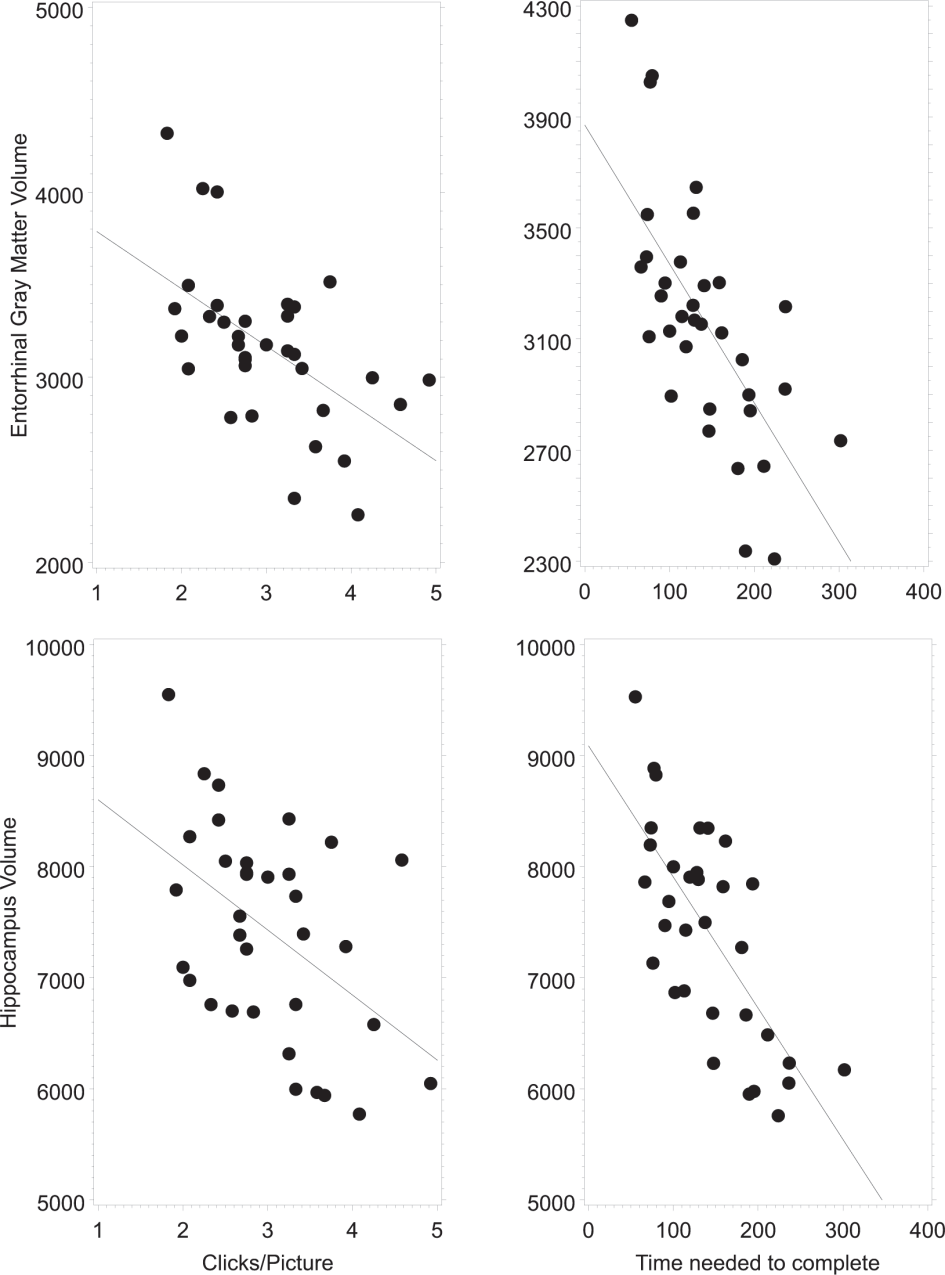
Correlations between the volume of the hippocampus, the volume of the entorhinal cortex, and the results of the memory game. Results of the memory game were assessed by the time needed to complete the game (panels on the left) and the trials or clicks needed to find a pair of shapes or pictures (panels on the right). Two upper panels: Volume of the Entorhinal Cortex. Two lower panels: Volume of the Hippocampus. Volumes are predicted from the General Linear Model analysis.

The number of trials to complete the memory game correlated with the volume of the hippocampus (R = -0.4, 95%CL = [-0.65 -0.04], n = 34, p = 0.03) (**Fig. 1**), the volume of the entorhinal cortex (R = -0.41, 95%CL = [-0.66 -0.05], n = 34, p = 0.02), and the volume of the temporal pole (R = -0.44, 95%CL = [-0.68 -0.09], n = 34, p = 0.01). Furthermore the time to complete the game correlated with the volume of the hippocampus (R = -0.54, 95%CL = [-0.75 -0.22], n = 34, p = 0.002) (**Fig. 1**), the volume of the entorhinal cortex (R = -0.42, 95%CL = [-0.67 -0.07], n = 34, p = 0.02), and the volume of the temporal pole (R = -0.54, 95%CL = [-0.75 -0.22], n = 34, p = 0.002).

### Correlations between Other CNS Structures and the Memory Game

There were no correlations between the number of trials and time needed to complete the memory game and the TIV, the Total Cortex Volume, and the amount of the Cerebrospinal Fluid (p>0.05). Analyses were corrected for age and gender.

### Correlations between Neuropsychological Tests, Clinical measures and the Memory Game

The adjusted number of total trials (R = 0.4, 95%CL = [0.05 0.66] n = 33, p = 0.03), the number of patterns correctly located after first trial (R = -0.42, 95%CL = [-0.67 -0.07] n = 33, p = 0.02), and the number of stages completed (Spearman R = -0.47, 95%CL = [-0.70 -0.13] n = 33, p = 0.008) in the PAL test correlated with the time needed to complete the memory game. There were correlations between the adjusted number of total trials (R = 0.37, 95%CL = [0.01 0.63], n = 33, p = 0.04), the number of stages completed (Spearman R = -0.39, 95%CL = [-0.65 -0.04], n = 33, p = 0.03) in the PAL test and the number of trials to complete the memory game. The number of patterns correctly located after first trial in the PAL test did not correlate with total number of trials to complete the memory game (R = -0.25, 95%CL = [-0.54 0.12], n = 33, p = 0.18). All analyses corrected for age and gender. The raw number of total trials in the PAL test did not correlate with the results of the memory game (p>0.05).

The time needed to complete the Trail Making test part A and B did not correlate with the results of the memory game (p>0.05). Results of the memory game did not correlate with the GDS and the STAI scores (p>0.05).

The ACE and the RAVLT total scores correlated with the time needed to complete the memory game, while the anterograde memory subscore of the ACE correlated with the trials needed to complete the memory game. The MMSE score and the delayed retrieval subscore of the RAVLT did not correlate with the memory game results (**Table 1**).

### Results from a pilot study: Differences between subjects with MCI and healthy controls in the memory game

Differences between study groups were analyzed by logistic regression (PROC LOGISTIC in SAS) with study group as response variable. Memory game results served as predictor variables in two separate analyses with age, and gender as covariates. We chose logistic regression, since it is an effective statistical method to compare the results of relative small study groups with at least 10 subjects in the smaller group, which is a rule of thumb in logistic regression according to the literature [45]. Healthy controls needed less trials (Chi-Square = 6; n = 46, p = 0.02, Odds Ratio = 2.9, 95% CI = 1.2–7.9, Sensitivity = 83%, Specificity = 62%, Cut-off = 4.5 trials/picture), and less time (Chi-Square = 6,2; n = 46, p = 0.01, Odds Ratio = 1.2, 95% CI = 1.1–1.5, Sensitivity = 82%, Specificity = 67%, Cut-off = 250sec) to complete the memory game than subjects with MCI. None of the covariates reached statistical significance (p>0.05).

## Discussion

Currently known preventive therapies and therapies to be introduced in the near future will likely be more effective in the early stage of dementia, when everyday functioning is not affected, i.e. when neuron loss is minimal [6,11]. Accordingly the identification of people forming a risk group with respect to later dementia may be particularly important.

Several recent studies have demonstrated that the volume of the temporal lobe structures such as the hippocampus can differentiate between healthy subjects, patients with MCI and patients with Alzheimer’s disease [46,47]. In addition, more and more studies have demonstrated that volumes of temporal structures predict the later development of Alzheimer’s disease in healthy subjects [48] and patients with MCI [49], in other words these brain volumes can identify subjects at risk before everyday functioning would be affected. In the present study the selection of the hippocampus, the entorhinal cortex and the temporal pole for validation was primarily based on the above evidence gathered during long-term follow-up investigations such as the Alzheimer's Disease Neuroimaging Initiative (ADNI) [23]. In addition to the above, multiple studies have underlined the importance of the entorhinal cortex in prediction of developing dementia in healthy subjects, and patients with MCI [47,48]. Furthermore the possibility of developing dementia later was significantly higher among those elderly persons whose decreased metabolism in entorhinal cortex was observed by FDG-PET imaging based on the data of a 3-year-long follow-up study including 48 persons [50].

### Results from the primary correlational analysis involving healthy subjects

The aim of the investigation was to show that widely used computer games can help to identify subjects at risk. In order to show this, analysis was conducted of the correlations between the results of the 'Find the Pairs' game and the volumes of those CNS structures previously found to be good predictors of later cognitive decline. The study found that subjects with smaller entorhinal cortex, temporal pole and hippocampus volumes needed more trials and more time to complete the memory game (**Fig. 1**). Thus, the results support the initial hypothesis that healthy individuals achieving worse results in the memory game have increased level of atrophy in the predefined brain structures by structural MRI. Additionally the study found no correlation between the results of the memory game and the total intracranial volume, the total cortex volume, and the amount of the cerebrospinal fluid, which shows the specificity of the above correlations. In other words this verifies that the above correlations are not due to general atrophy rather a consequence of a more specific process starting in the temporal regions.

In addition to brain imaging technologies, the predictive strength of neuropsychological tests has also been demonstrated by several studies [5,6]. In the present study the PAL Test was included to assess associative learning between visual stimuli and different spatial locations [44]. The effectiveness and predictive strength of this test in the early diagnosis of dementia was shown by several follow-up studies [6,7]. We found that the subjects who completed the memory game faster, and from fewer trials, could competed more stages and more patterns from first trial, and needed less trials in the PAL test. The results of the Trail Making Test part A and B, assessing mainly selective attention and executive functions respectively, did not correlate with the game results, which indicate that decreased performance in the memory game is not part of a general cognitive slowing.

Furthermore it was found that better results in the memory game are also associated with the higher ACE total scores and anterograde memory subscores. Previous investigations showed that the ACE is a useful tool to predict later conversion to dementia [51]. The MMSE was used for the assessment of global cognitive functions and the RAVLT was used for the detailed investigation of the short term memory. Since exclusion of subjects with MCI and dementia was based on these tests, no correlation was expected with the results of memory game in these tests due to the small variance in the healthy subject group. Despite the low variance the results indicate that higher RAVLT total scores were associated with better results in the memory game. No correlation was found between the results of STAI and GDS and the results of the memory game indicating that decreased performance was not caused by anxiety or depression.

### Differences between subjects with MCI and healthy controls in the memory game

A significant difference was found between the study groups in the trials needed to complete the memory game showing that it can differentiate between subjects with MCI and healthy controls with a moderate sensitivity (>80%) and a low specificity (>60%). A limitation of this finding is the low number of subjects in the MCI groups.

### Conclusions

In summary the results support the initial hypothesis that healthy elderly subjects achieving worse results in the memory game have increased level of atrophy in the temporal brain structures and showed a decreased performance in the PAL test, in the ACE, and in the RAVLT, which were previously extensively validated and considered to be sensitive tools in dementia prediction. Furthermore subjects with MCI achieved significantly worse in the memory game compared to healthy controls. Based on these results it can be concluded that memory games such as the 'Find the Pairs' game may be useful in detecting the early signs of cognitive decline.

However, the evidence gathered in the present investigation is indirect, since the study was cross-sectional, and hippocampus is a promising, but only surrogate biomarker, therefore our results should be confirmed by long term follow-up studies in the future. For the same reason test-retest reliability of the memory game cannot be reported. A further limitation of this investigation was the relative small sample size.

Compared with MRI and neuropsychological testing, the benefits of computer games are their accessibility, their cost-effectiveness, and the involvement of healthy subjects, or subjects with MCI. Furthermore cognitive games can be played at home, where anxiety caused by the clinical environment is not present and does not reduce performance. However their lower sensitivity and specificity are definite drawbacks. Therefore they can be used to give feedbacks to the players, and may give a hint to them that it might be useful to seek professional help. This may help people to seek help in the beginning of the cognitive decline far earlier than they currently do. It must be emphasized toward the users, that these computer games are not appropriate for diagnosing and therefore they cannot replace the detailed neuropsychological investigation in clinical practice. However the findings of this study support the idea that such games can help people at risk to seek professional help in time.

## Supporting Information

S1 Table Raw data in excel format. (XLSX)
